# Supplementary material for: CryoEM structure of the SLFN14 endoribonuclease reveals insight into RNA binding and cleavage
Source: Nat Commun. 2025 Jul 1;16:5848. doi: 10.1038/s41467-025-61091-8 (PMC12215978; doi:10.1038/s41467-025-61091-8)
Supplement: Supplementary file 1 — Supplementary Information [file 41467_2025_61091_MOESM1_ESM.pdf]

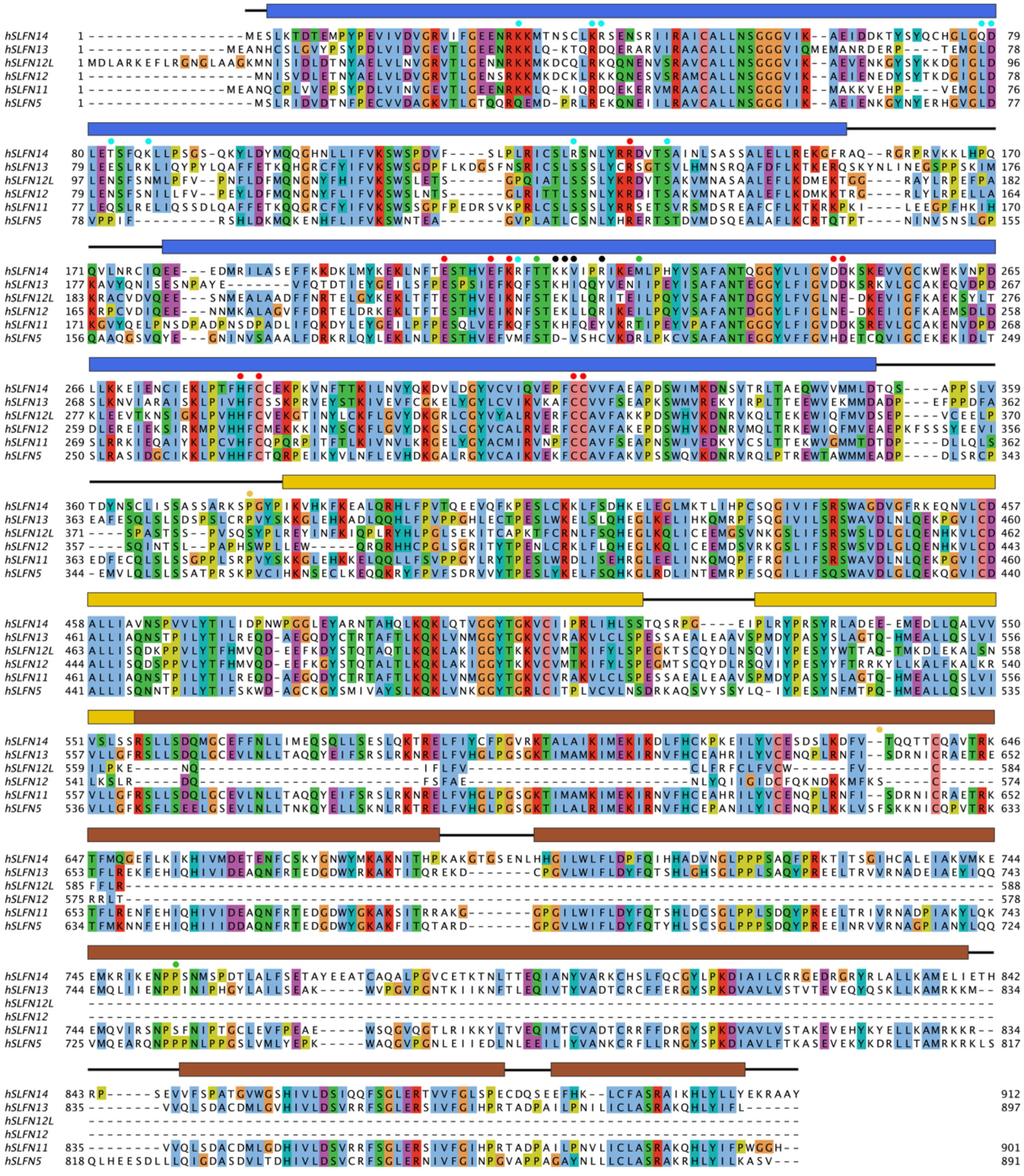

Supplementary Figure 1. Multiple amino acid sequence alignment of the SLFN family. Sequence alignment of the *Homo sapiens* (h) SLFN family members SLFN14, SLFN13, SLFN12L, SLFN12, SLFN11, and SLFN5. Alignments were performed with PROMALS3D[1] and illustrated by JalView 2.11.4.1[2]. Above the alignment are the observed structured regions colored in blue, yellow, and brown to designate the SLFN14 N-terminal domain, middle domain, and C-terminal domain, respectively. The black line represents unstructured regions not modeled in the SLFN14•RNA structure. SLFN14 residues associated with inherited thrombocytopenia (K218N, K219E/N, V220D, R223W)[3, 4, 5] are marked by black dots, SLFN11 phosphorylated residues[6] are demarcated by green dots, SLFN12 phosphorylated residues[7] are marked by yellow dots, residues at the N- and C-lobe RNA binding interfaces are cyan dots, and residues mutated in this study are indicated by red dots.

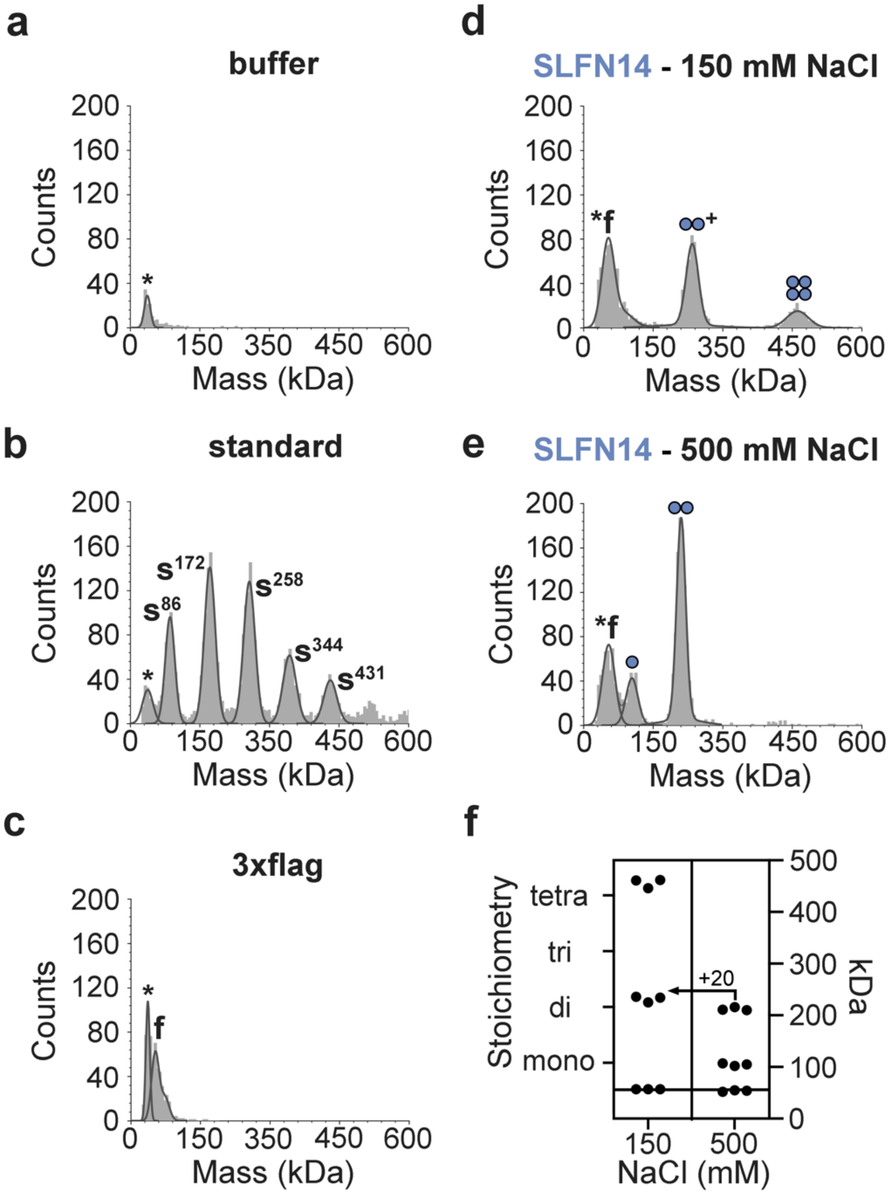

Supplementary Figure 2. Mass distribution of SLFN14 protein. Mass photometry analysis of **a** storage buffer, **b** P1 calibrant, **c** 3x flag peptide, **d** 25 nM SLFN14 protein in 150 mM NaCl storage buffer, and **e** 25 nM SLFN14 protein in 500 mM NaCl storage buffer with total counts on the y-axis and mass (kDa) on the x-axis. **f** Distribution of SLFN14 stoichiometry (primary y-axis) and mass (secondary y-axis) in low and high salt storage buffer (y-axis). Arrow highlights the molecular mass differential of the SLFN14 dimer between three replicates measured in low and high salt conditions. +20 defines the calculated average mass differential and accompanying standard deviation (20 ± 6.71 kDa) between dimer species at low and high salt. n=3 samples/independent measurement. Source data are provided as a Source Data file.

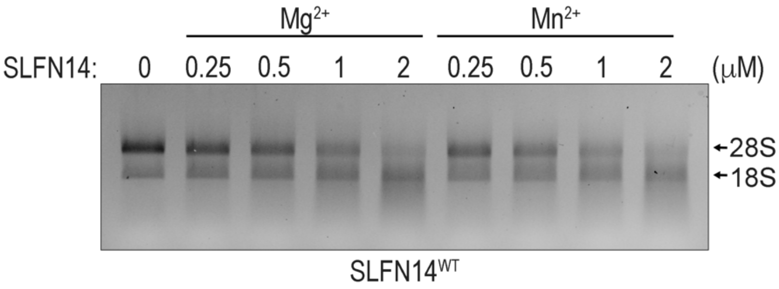

Supplementary Figure 3. Characterization of SLFN14 rRNA cleavage in the presence of Mg^{2+} and Mn^{2+} ion. Representative in vitro rRNA cleavage assay of SLFN14 wild-type variant incubated with isolated total human RNA (1 μg) and Mg^{2+} metal ion containing reaction buffer supplemented with either 1 mM Mg^{2+} or Mn^{2+} ion. Mammalian 28S and 18S rRNA species are identified by black arrows. n=3 samples/independent experiment. Source data are provided as a Source Data file.

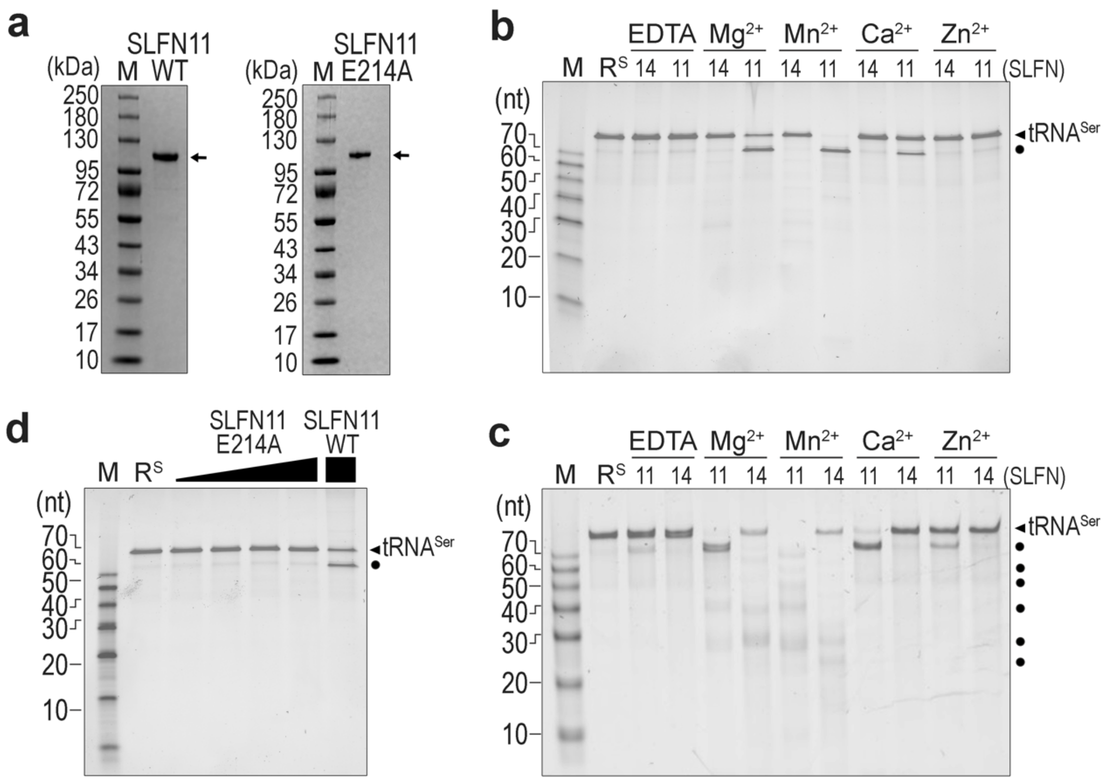

Supplementary Figure 4. Biochemical characterization of SLFN14 and SLFN11 RNA cleavage. a SDS-PAGE analysis of human SLFN11 WT (left) and E214A catalytic mutant (right). Arrows mark the expected migration of full-length protein. Gel was visualized by Coomassie blue stain. **b** Denaturing urea-PAGE of synthetic tRNA^{Ser} substrate (100 nM) incubated with 50 nM SLFN protein. The reaction buffer contains 1 mM Mg^{2+} metal ion and is supplemented with 1 mM metal ion or EDTA, as shown above the gel. **c** RNA cleavage reaction described in panel b with 200 nM SLFN protein. **d** Denaturing urea-PAGE of synthetic (R^S) tRNA^{Ser} substrate (100 nM) incubated in the absence and presence of SLFN11 variants. Black triangle marks a titration of the SLFN11 E214A variant (50, 100, 200, and 400 nM) and WT marks a reaction where synthetic (R^S) tRNA^{Ser} is incubated with 400 nM SLFN11 wild-type protein. The black arrowhead marks the unprocessed tRNA^{Ser} substrate and circles mark cleaved RNA product. M defines the 70-10-nt RNA ladder with the corresponding nucleotide length on the left. n=3 samples/independent experiment. Source data are provided as a Source Data file.

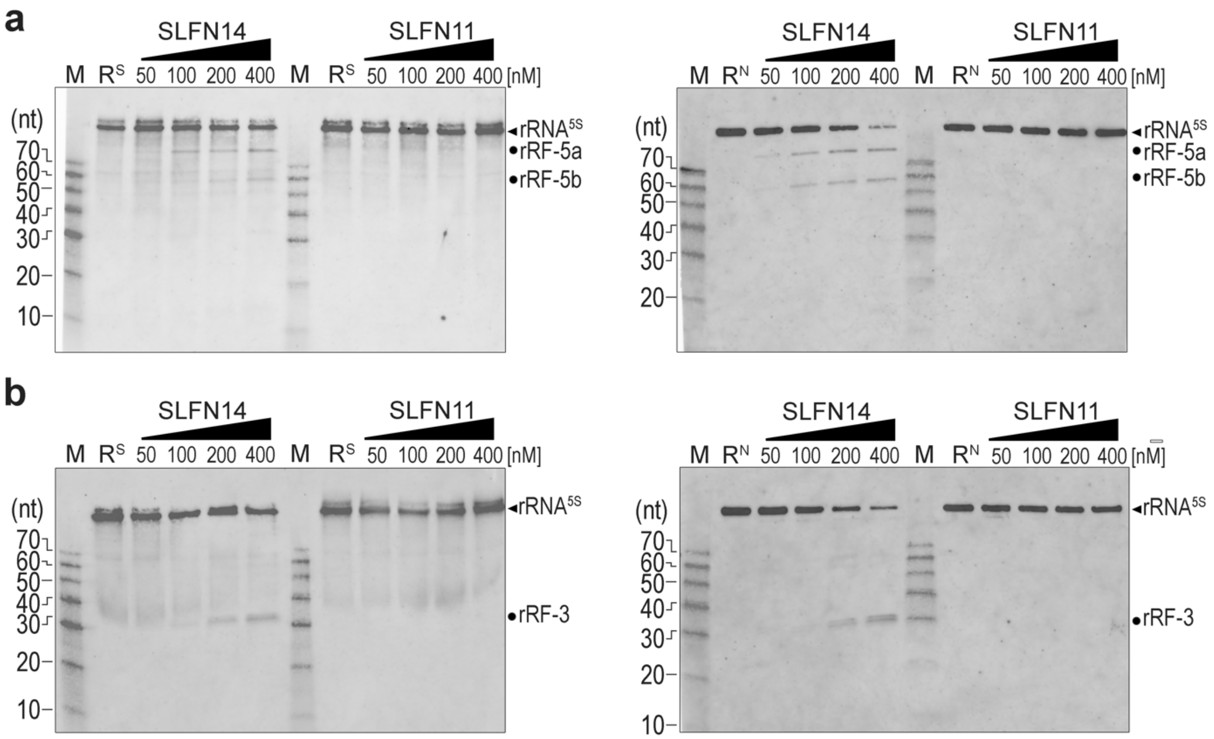

Supplementary Figure 5. Cleavage of 5S rRNA substrates by human SLFN14 and SLFN11. Representative SLFN titrations (black triangles) with protein concentrations shown above (50, 100, 200, and 400 nM). Northern blots of denaturing urea-PAGE gels are shown for the cleavage of synthetic (R^S, 100 nM) and native (R^N, 1 μM total human small RNA) 5S rRNA visualized using: **a** 5′-targeting 5S rRNA probe and **b** 3′-targeting 5S rRNA probe. M defines the 70-10-nt RNA ladder with the corresponding nucleotide length on the left. The black arrowhead marks the unprocessed 5S rRNA substrate ($rRNA^{5S}$), and the black dots mark cleaved 5′- and 3′-rRNA fragments (rRF-5 and rRF-3), respectively. Distinct 5′ rRNA fragments are further distinguished by the letter a and b. n=3 samples/independent experiment. Source data are provided as a Source Data file.

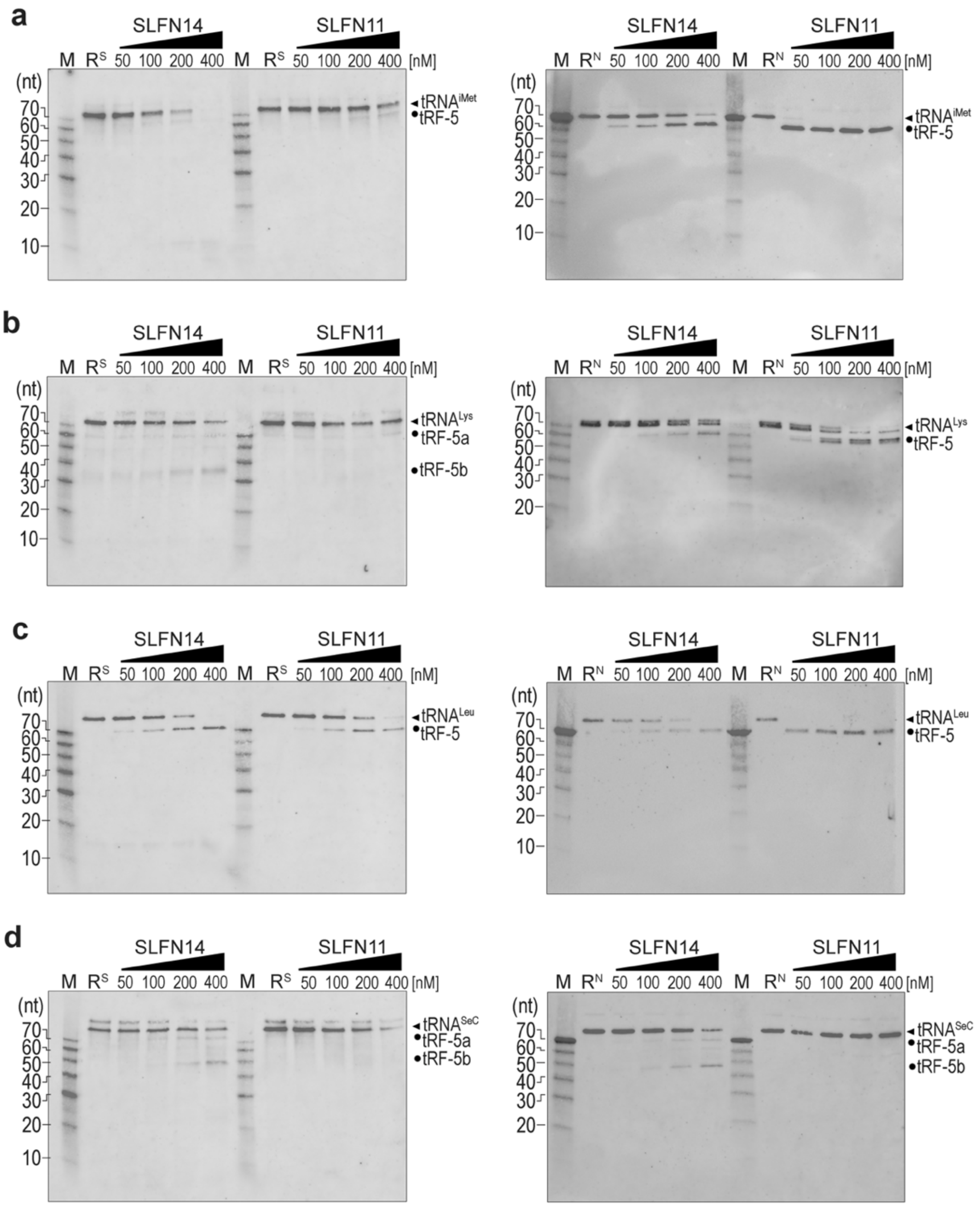

Supplementary Figure 6. Cleavage of tRNA substrates by human SLFN14 and SLFN11. Representative SLFN titrations (black triangles) with protein concentrations shown above (50, 100, 200, and 400 nM). Northern blots of denaturing urea-PAGE gels are shown for the cleavage of synthetic (R^S, 100 nM) and native (R^N, 1 μM total human small RNA) **a** $tRNA^{iMet}$ substrate, **b** $tRNA^{Lys}$ substrate, **c** $tRNA^{Leu}$ substrate, and **d** $tRNA^{SeC}$ substrate. M defines the 70-10-nt RNA ladder with the corresponding nucleotide length on the left. The black arrowhead marks the unprocessed tRNA substrate (tRNA) with the codon identity superscript, and the black dots mark cleaved 5′-tRNA fragments (tRF-5). Distinct 5′ tRNA fragments are further distinguished by the letter a and b. n=3 samples/independent experiment. Source data are provided as a Source Data file.

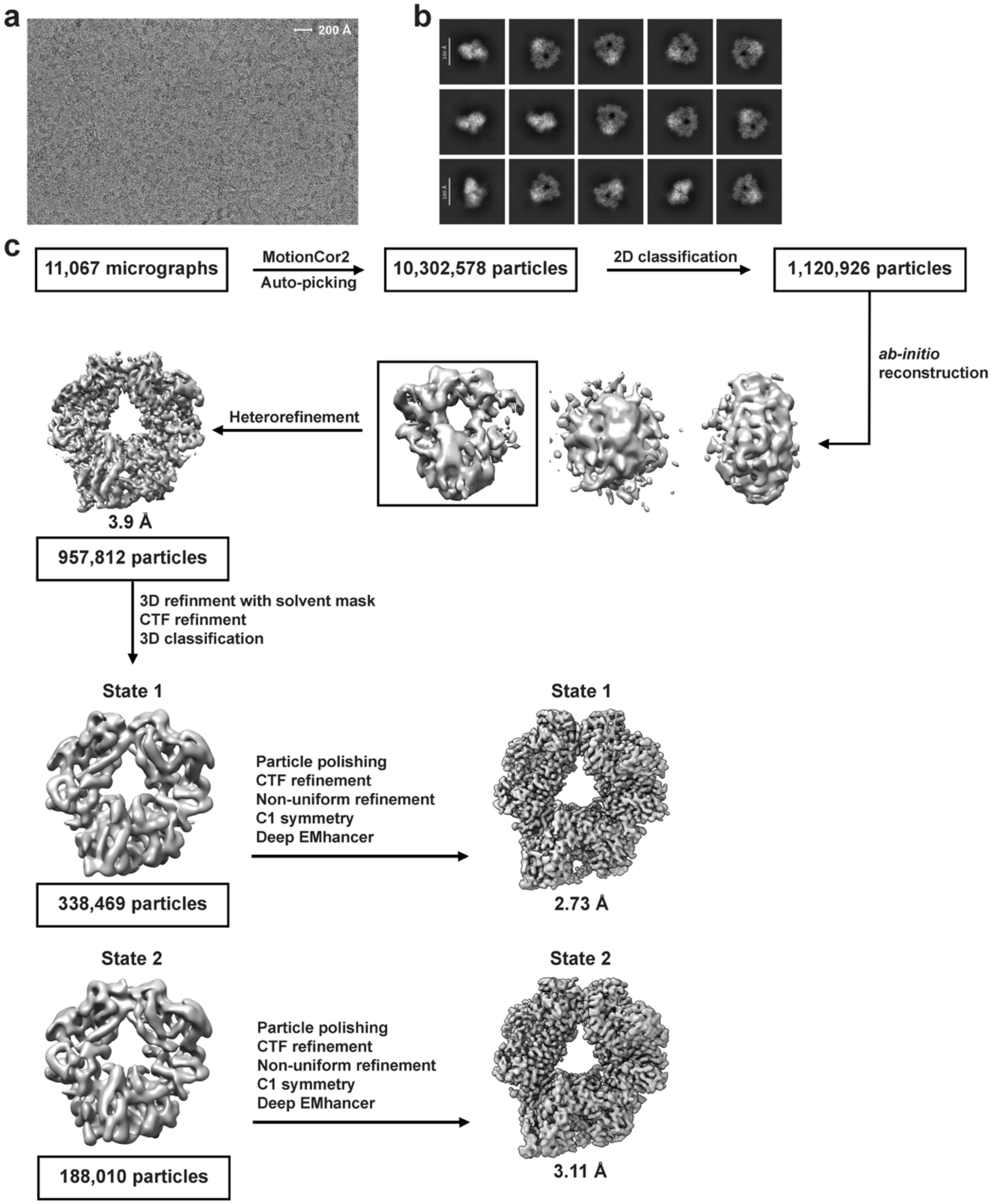

Supplementary Figure 7. Overview of cryoEM processing scheme for SLFN14•RNA. a A representative micrograph of SLFN14 in vitreous ice from a total of 11,067 micrographs. **b** Select 2D classes generated from 11,067 movies collected from 200-mesh QUANTIFOIL R1.2/1.3 2 nm ultra-thin carbon grids. **c** CryoEM processing workflow. Particles picked (10,302,578) were subjected to 2D classification, 3D classification, and refinement in cryoSPARC v4.2.1 and v4.6.2[8] and RELION 4.0[9]. The 2.73 Å cryoEM reconstruction was used to build a model of RNA-bound SLFN14.

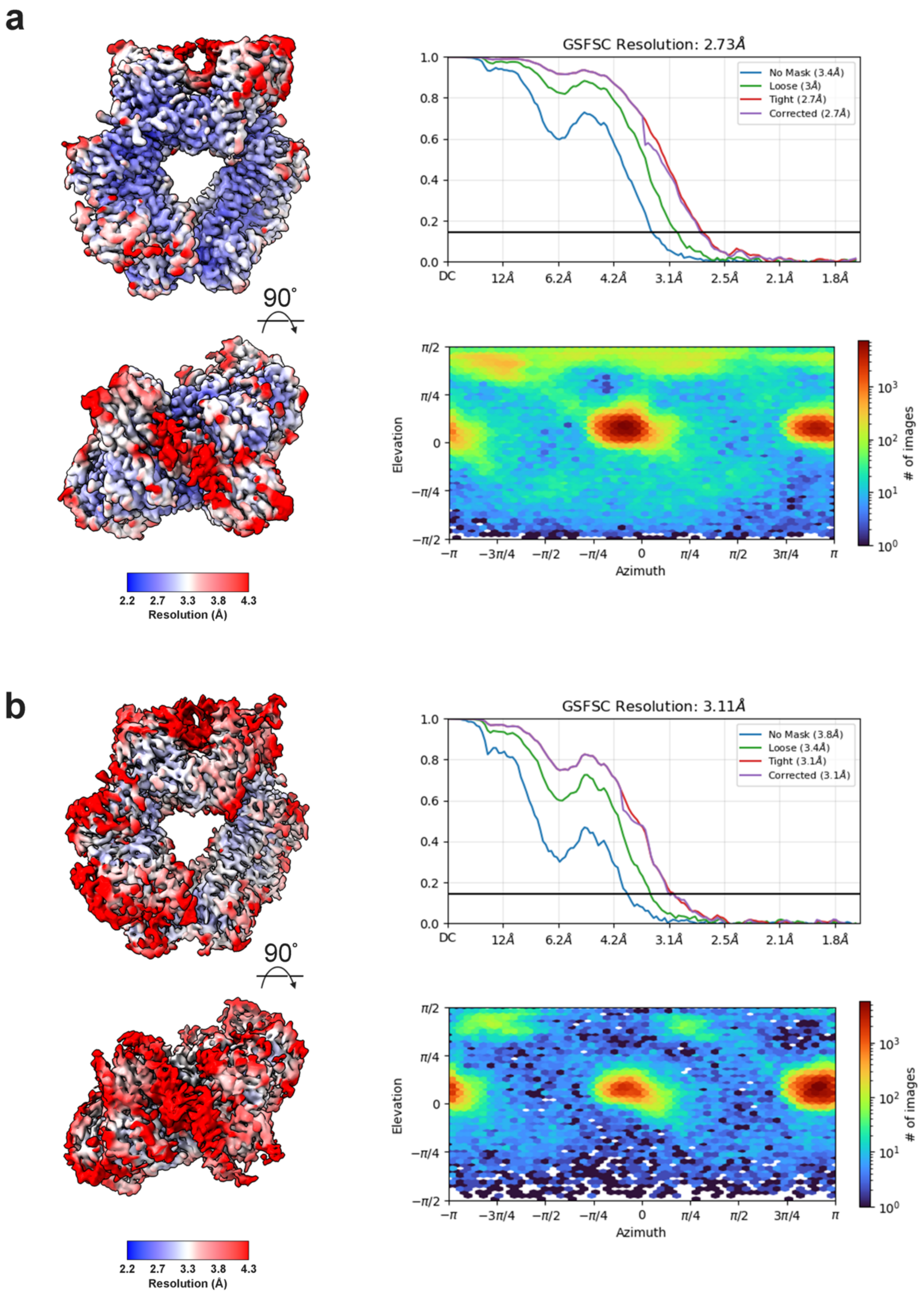

Supplementary Figure 8. Validation of the SLFN14•RNA reconstructions. Local resolution of cryoEM reconstructions determined by Resmap[10], standard Fourier Shell Correlation (FSC) curves, and angular distribution of SLFN14 particles for **a** state 1 cryoEM reconstruction and **b** state 2 reconstruction using cryoSPARC v4.2.1 and v4.6.2.

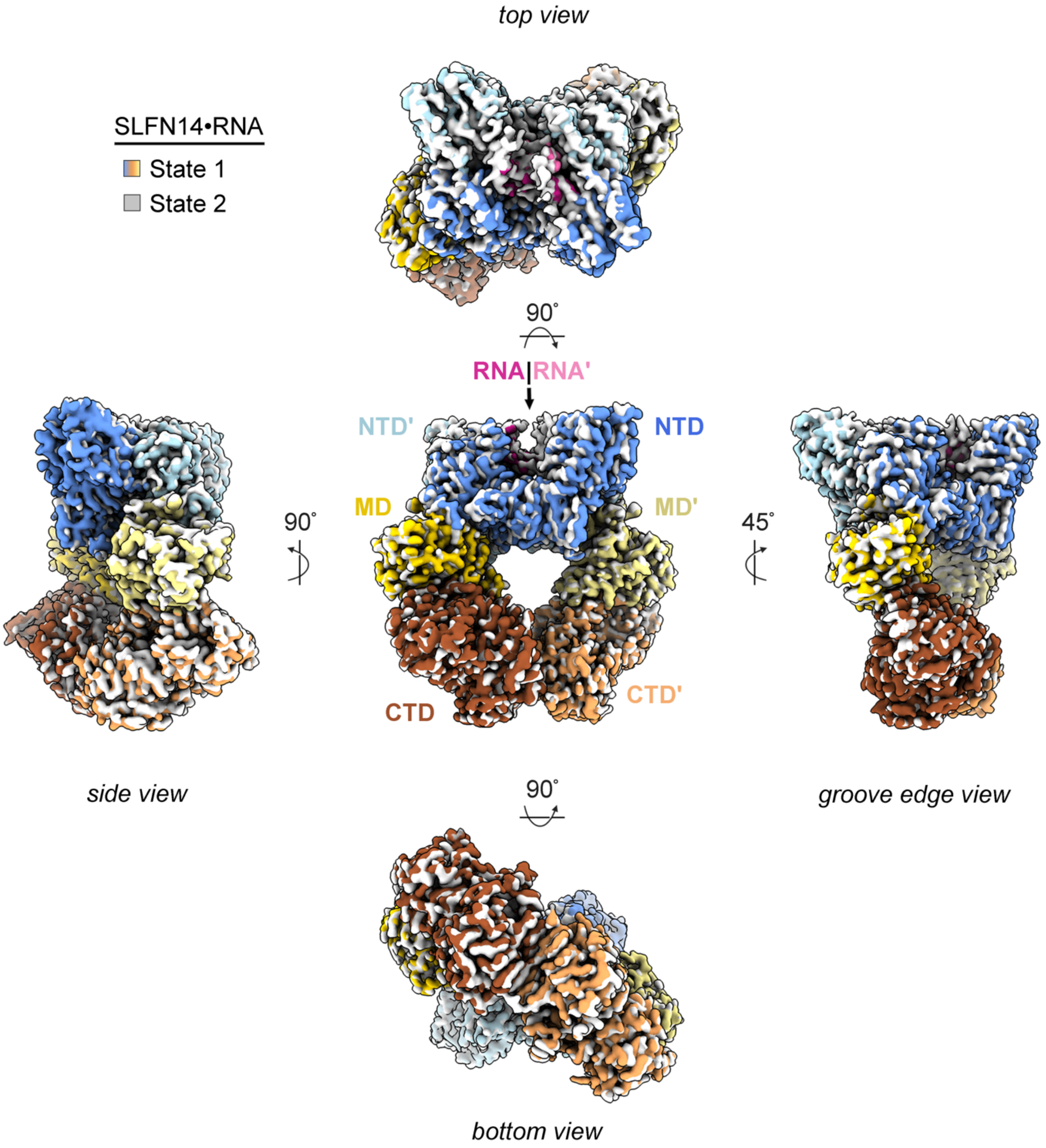

Supplementary Figure 9. Conformational heterogeneity of SLFN14 N- and C-terminal domains. Overlay of state 1 (colored) and state 2 (grey) of the SLFN14•RNA cryoEM reconstructions to illustrate the conformational heterogeneity in the N-terminal and C-terminal domains. The SLFN14 N-terminal, middle, and C-terminal domains are colored blue, yellow, and brown. Dimeric SLFN14 protomers are distinguished in dark and light colors. Prime symbol demarcates protomer B.

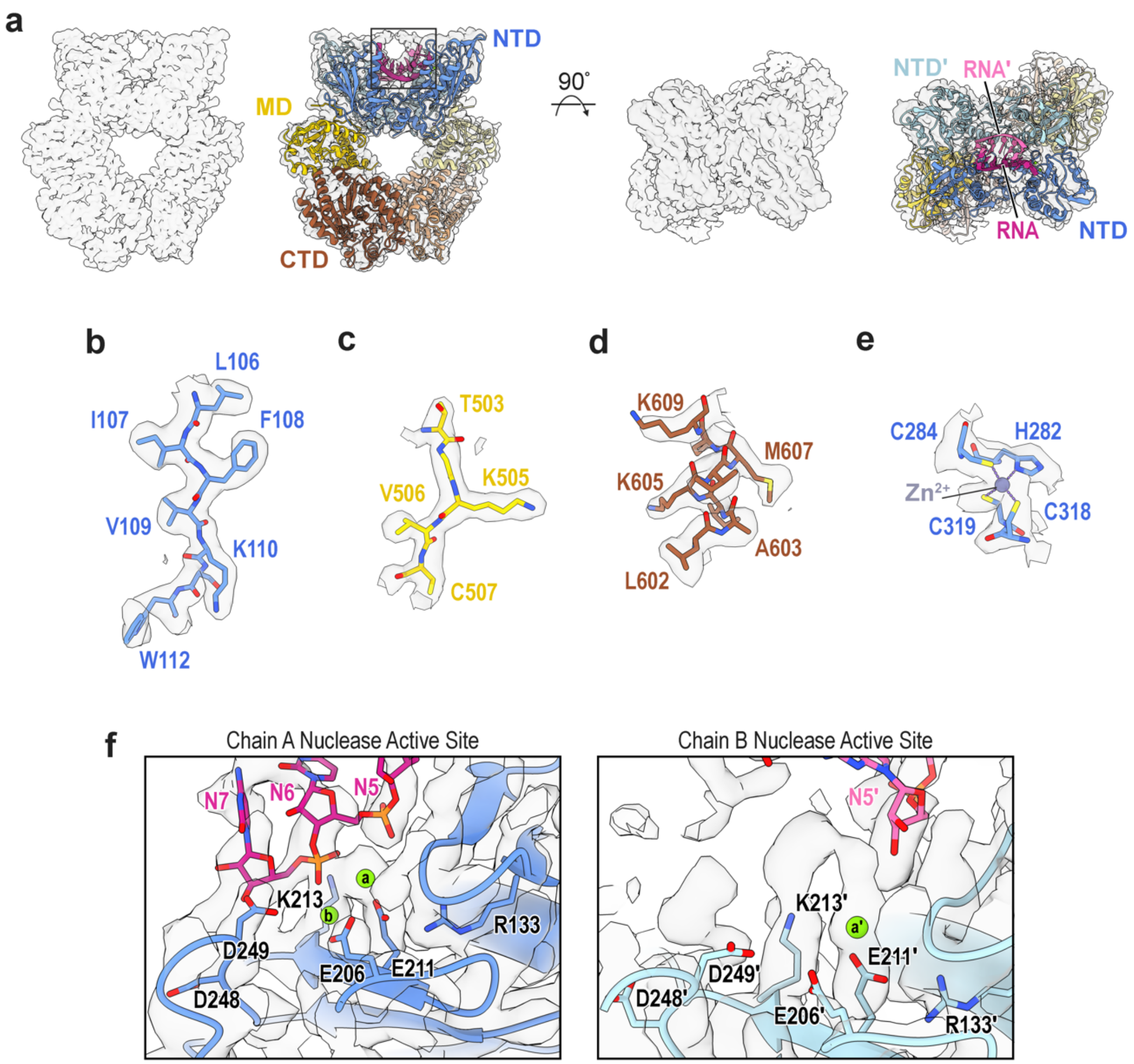

Supplementary Figure 10. CryoEM reconstruction with SLFN14•RNA cartoon overlay. Representative state 1 cryoEM reconstruction and corresponding model for protomer A of **a** SLFN14•RNA, **b** N-terminal domain, **c** middle domain, **d** C-terminal domain, and **e** Zn^{2+} metal ion binding site. **f** The Mg^{2+} metal ion binding site along with nearby catalytic residues and model RNA are shown for SLFN14 protomer A (left) and protomer B (right). The SLFN14 N-terminal, middle, and C-terminal domains are colored blue, yellow, and brown, respectively. RNA strands are depicted as dark and light pink. Mg^{2+} and Zn^{2+} metal ions are shown as green and grey spheres, respectively. Prime symbol demarcates protomer B.

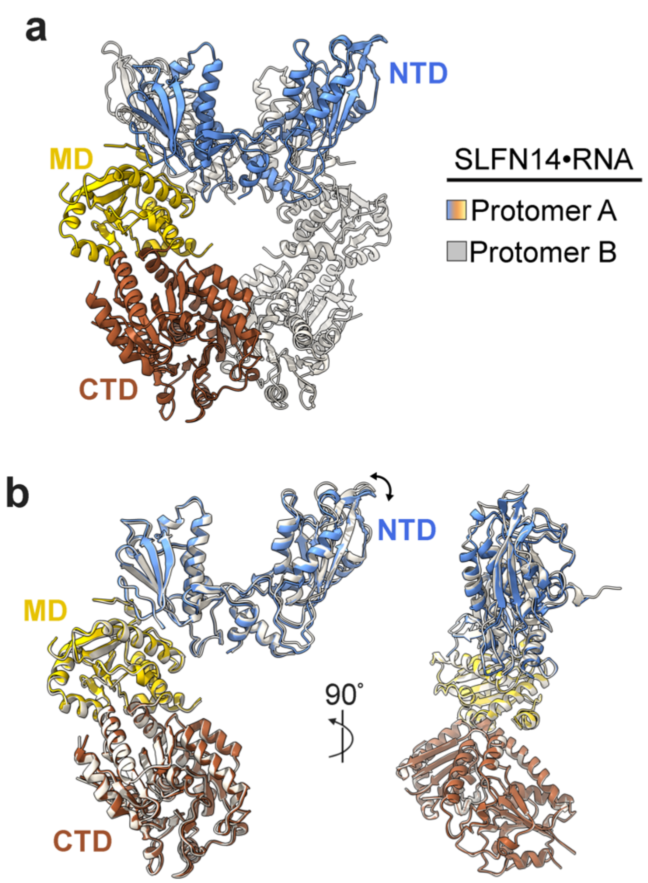

Supplementary Figure 11. Overlay of state 1 SLFN14 protomers A and B. a Cartoon representation of the state 1 SLFN14 homodimer. SLFN14 protomer A is colored and protomer B is in grey. **b** Superimposition of protomers A and B as colored in panel a. Double headed arrow highlights the conformational change between protomers within the C-lobe of the NTD.

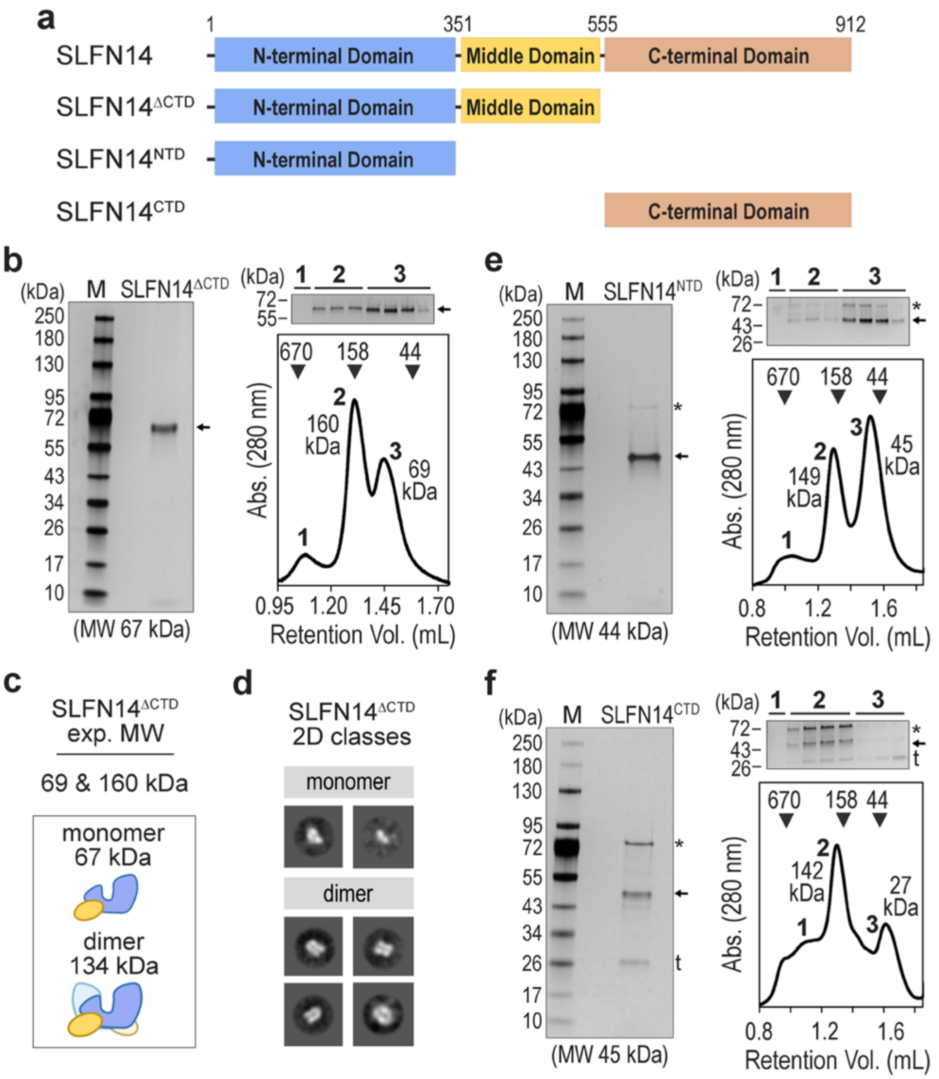

Supplementary Figure 12. SLFN14 CTD facilitates homodimerization. a Cartoon diagram of human SLFN14 variants with the numbering defining the amino acid domain boundaries. The SLFN14 N-terminal domain is shown in blue, the middle domain is colored yellow, and the C-terminal domain is depicted as brown. **b** SDS-PAGE analysis of purified recombinant human SLFN14$^{\Delta CTD}$. Gel was visualized by silver stain, and the theoretical molecular weight of SLFN14$^{\Delta CTD}$ is shown in brackets. Size-exclusion chromatography of SLFN14$^{\Delta CTD}$ with Absorbance at 280 nm on the y-axis and retention volume on the x-axis. Molecular weight (kDa) standards are indicated at the top of the plot. SDS-PAGE analysis of peaks 1 and 2 are shown above and visualized by silver stain. n=2 samples/independent experiment. **c** SLFN14$^{\Delta CTD}$ calculated molecular weight species determined by size-exclusion chromatography as shown in panel b. Cartoon depiction of monomeric and dimeric SLFN14$^{\Delta CTD}$ and their corresponding theoretical molecular weights. **d** Select reference-free 2D classes of monomeric and dimeric SLFN14$^{\Delta CTD}$. **e** Biophysical analysis of SLFN14^{NTD} as described in panel b. Asterisk marks an unknown contaminant. n=2 samples/independent experiment. **f** Biophysical analysis of SLFN14^{CTD} as described in panel b. The asterisk marks an unknown contaminant and t identifies a possible SLFN14^{CTD} truncation. M defines a protein ladder with units in kilodalton (kDa). n=2 samples/independent experiment. Source data are provided as a Source Data file.

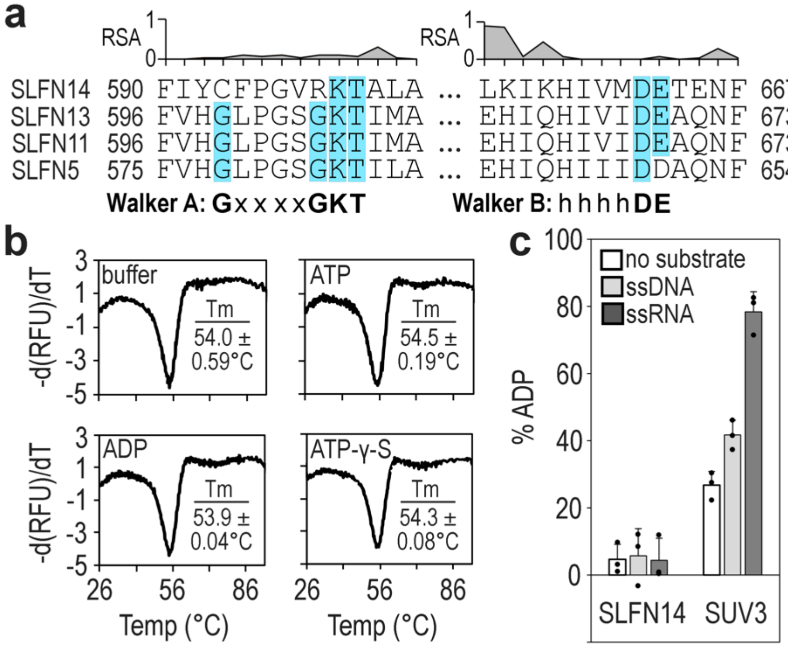

Supplementary Figure 13. The SLFN14 dimer harbors an occluded ATPase site. a Multiple amino acid sequence alignment of conserved Walker A and Walker B motifs of human SLFN family members. Conserved residues of the consensus Walker A (GxxxxGKT) and Walker B (hhhhDE) motifs are highlighted in blue[11]. Alignments were performed with PROMALS3D[1], and relative surface accessibility (RSA, y-axis) was calculated with NetSurfP-3.0[12] and plotted for individual Walker A and B residues (x-axis). **b** Thermal stability of SLFN14 (1 μM) in the absence (buffer) and presence of ATP, ADP, or ATPγS nucleotide (1 μM). The first derivative of the change in relative fluorescence unit (-d(RFU)/dT) is on the y-axis and temperature in degree Celsius (°C) is shown on the x-axis. The average melting temperature (Tm) and standard deviation were calculated from n=3 samples/independent experiment. **c** ATPase activity of human SLFN14 (2 μM) and confirmed ATPase human SUV3 (2 μM)[13] with 5 μM ATP in the absence and presence of ssRNA and ssDNA substrates (2 μM). The percent ADP is shown on the y-axis and the average ADP percentage is represented as a bar with error bars defined as the standard deviation from n=3 samples/independent experiment. Black dots are displayed for each individual replicate measurement. Source data are provided as a Source Data file.

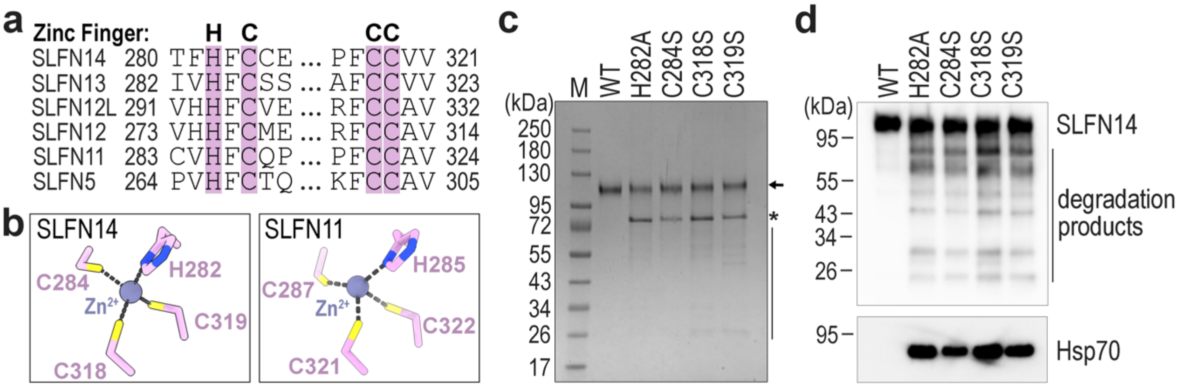

Supplementary Figure 14. The SLFN14 zinc finger is a structural motif. a Conserved zinc finger motif of the human SLFN family members. Conserved residues contributing to a canonical zinc finger motif are highlighted in purple. Alignments were performed with PROMALS3D[1]. **b** Zinc finger of human SLFN14 and human SLFN11 (PDB 7ZEL[14]). Residues that make up the conserved SLFN zinc finger motif are shown in purple, and the Zn^{2+} metal ion is shown as a grey sphere. **c** SDS-PAGE analysis of purified recombinant wild-type (WT) SLFN14 and zinc finger variants. Gel was visualized by SimplyBlue safe stain. Prominent and minor unidentified protein bands unique to the SLFN14 zinc finger variants are marked by an asterisk and black line, respectively. M defines a protein ladder with units in kilodalton (kDa). **d** Representative Western blot analysis of purified samples of SLFN14 wild-type (WT) and zinc finger variants. Zinc finger mutations result in the accumulation of SLFN14 degradation products (black line) and the co-purification of mammalian Hsp70. n=3 samples/independent experiment. Source data are provided as a Source Data file.

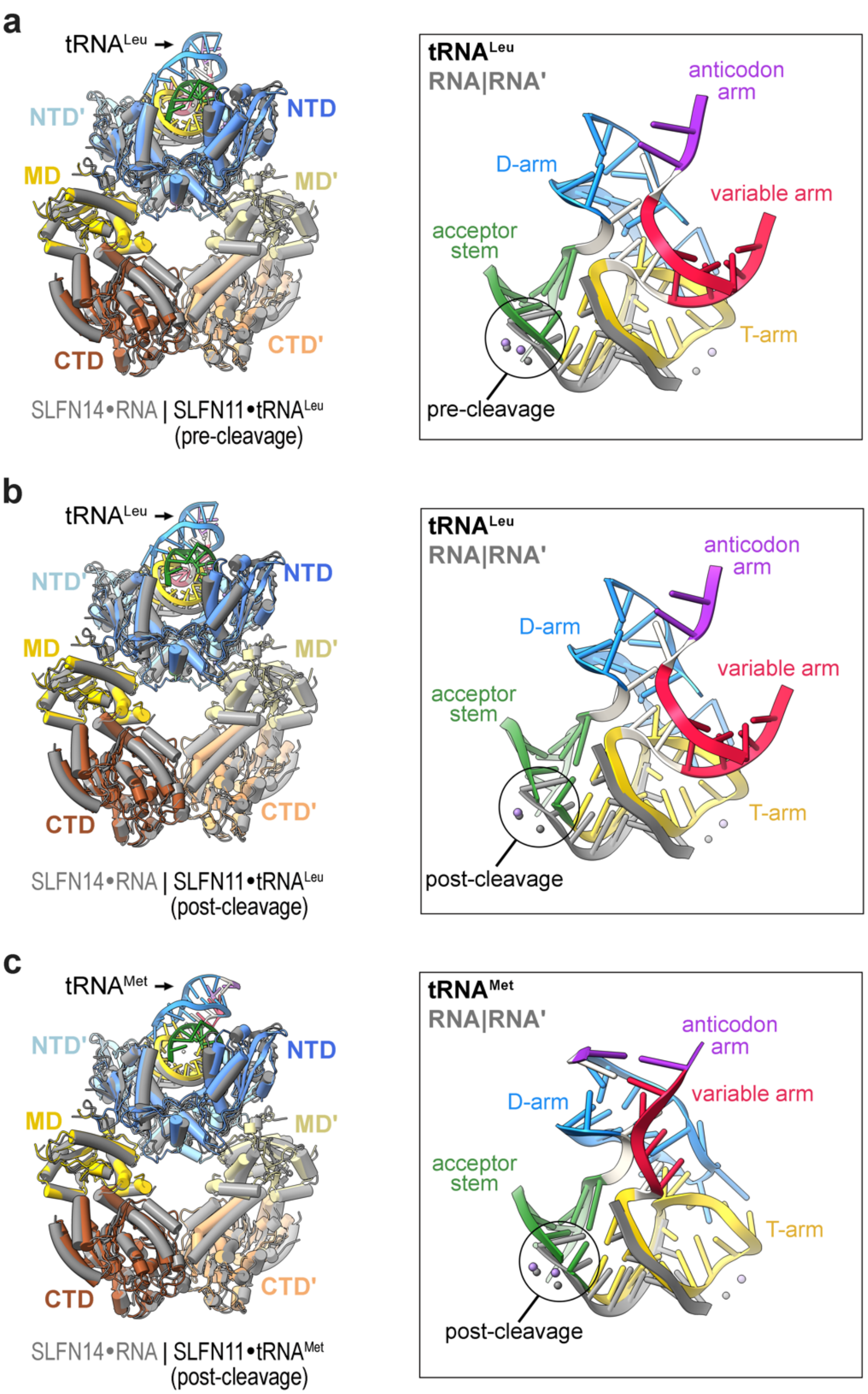

Supplementary Figure 15. Cartoon depiction of human SLFN14•RNA (dark grey) superimposed with pre-cleavage state of tRNA^{Leu}-bound SLFN11 (color, PDB 9GMW[15]). Inset illustrates overlay of RNA bound by SLFN14 and tRNA^{Leu} bound by SLFN11. The tRNA acceptor stem (green), D-arm (blue), anticodon arm (purple), variable arm (red), and T-arm (yellow) are colored. **b** As described in panel a with post-cleavage state of tRNA^{Leu}-bound SLFN11 (PDB 9GMX[15]). **c** Overlay as in panel a with post-cleavage state of tRNA^{Met}-bound SLFN11 (colored, PDB 9ERF[15]).

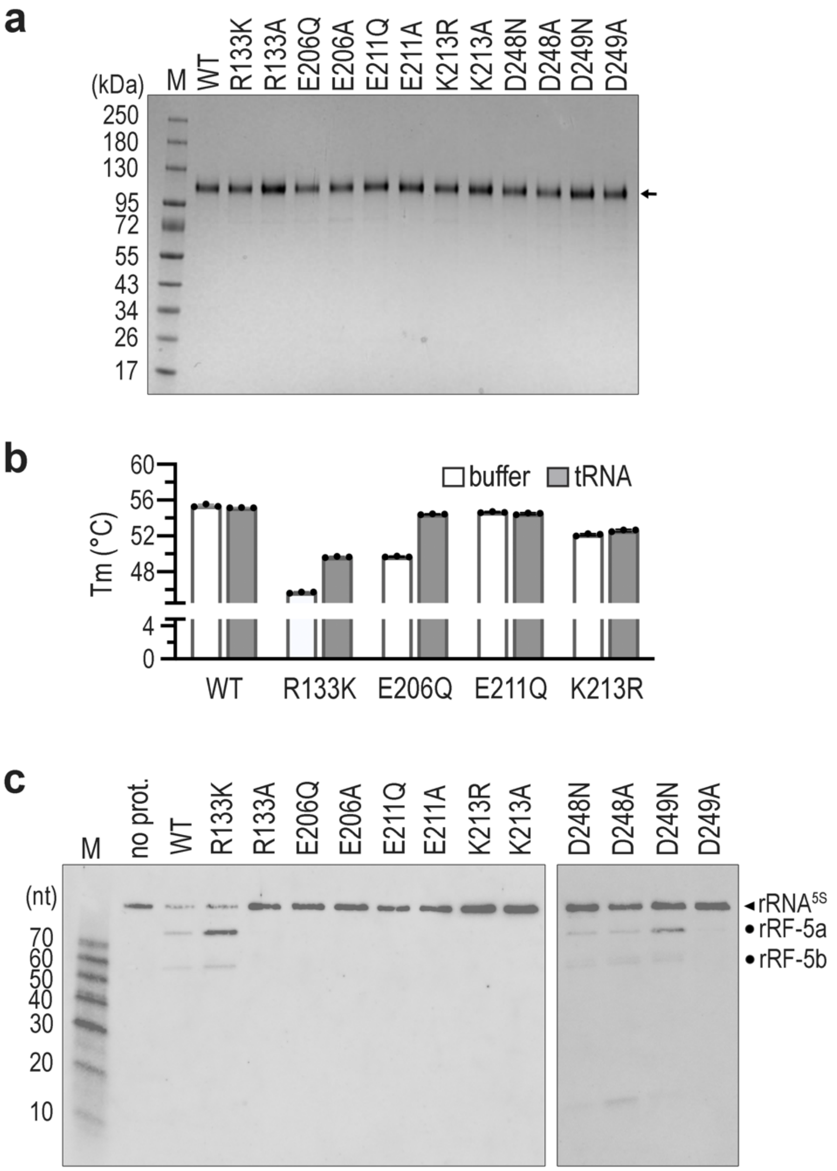

Supplementary Figure 16. Molecular characterization of SLFN14 C-lobe variants. a SDS-PAGE analysis of purified recombinant SLFN14 C-lobe variants. Gel was visualized by SimplyBlue safe stain. The arrow marks the expected migration of full-length SLFN14. M defines a protein ladder with units in kilodalton (kDa). **b** Thermal stability of SLFN14 C-lobe variants in the absence (white) and presence of native tRNA from brewer's yeast (grey). Melting temperature in degree Celsius (°C) is shown on the y-axis and SLFN14 variants are on the x-axis. The reported melting temperature (Tm) is the average of three independent measurements and error bars define the standard deviation. Black dots mark measurements from n=3 samples/independent experiment. **c** Representative Northern blot of native 5S rRNA (1 μM total human small RNA) cleavage by SLFN14 C-lobe variants (400 nM). M defines the 70-10-nt RNA ladder with the corresponding nucleotide (nt) length on the left. The black arrowhead marks the unprocessed 5S rRNA substrate ($rRNA^{5S}$), and the black dots mark cleaved 5′ 5S rRNA fragments (rRF-5) with letters a and b demarcating distinct fragments. n=3 samples/independent experiment. Source data are provided as a Source Data file.

Supplementary Methods

Molecular Cloning of SUV3 Protein Expression Vector

Bacterial expression plasmid of codon-optimized human SUV3 helicase (residues 46-786) with an upstream 6xHis-TEV sequence was synthesized and inserted into pETDuet-1 between NcoI and BamHI by GenScript (Piscataway, NJ). SUV3 residues 1-45 were omitted from the gene construct to mimic the post-proteolytic protein product generated upon removal of the mitochondrial targeting signal. Plasmid was verified by DNA sequencing. Please refer to Supplementary Data 1 for the expression plasmid used in this study.

SUV3 Helicase Expression and Purification

SUV3 was overexpressed in *Escherichia coli* Rosetta 2 (DE3) pLacI cells (Sigma, catalog: 71404-3), induced with 0.5 mM isopropylthio-β-galactoside (UBPBio, catalog: P1010), and incubated overnight at 18°C. Cells harboring recombinant SUV3 were resuspended in lysis buffer (50 mM Tris pH 8.0, 1 M NaCl, 10 mM imidazole, 1% Triton-X100). Resuspended cells were lysed by sonication and clarified at 26,916 x g for 50 min at 4°C. Clarified lysate was applied to a gravity flow column loaded with His60 Ni superflow resin (Takara, catalog: 635662), washed with lysis buffer supplemented with 30 mM imidazole, and eluted with elution buffer (50 mM Tris pH 8.0, 500 mM NaCl, 300 mM imidazole). SUV3 was resolved over a HiLoad 16/600 Superdex-200 Prep Grade (Cytiva) gel filtration column equilibrated with storage buffer (20 mM Hepes pH 7.8, 300 mM NaCl, 2 mM DTT). SUV3 was concentrated using a 10,000 NMWL centrifugal filter (Sigma, catalog: UFC901024) and flash-frozen for long-term storage at -80°C.

Differential Scanning Fluorimetry

Thermal denaturation curves of SLFN14 variants were generated by differential scanning fluorimetry. SLFN14 variants (1 μM) were incubated in the absence and presence of 2 μM tRNA

purified from brewer's yeast (Thermo Scientific, catalog: AM7119) and ATP, ADP, and ATPγS nucleotides (1 μM). Protein mixtures were prepared with 20x SYPRO orange protein gel stain (Invitrogen, catalog: S6651) in 50 mM Tris pH 7.4, 100 mM NaCl, 1 mM $MgCl_2$, 1 mM TCEP, and 2% glycerol. SLFN14 mixtures were incubated at 25°C for 30 minutes prior to applying a temperature gradient to 95°C at a rate of 0.96°C per minute. The fluorescent signal was measured on a QuantStudio 7 Flex Real-Time PCR System (Thermo Scientific) using QuantStudio Real-Time PCR software version v1.3. The melting temperature was determined using Protein Thermal Shift Software v1.3 (Applied Biosystems). Three technical replicates were performed to derive an average melting temperature and standard deviation. Unprocessed data of the differential scanning fluorimetry graphs are shown in the Source Data file.

ATP Hydrolysis Assay

Following the manufacturer's instructions, ATP hydrolysis activity was measured using the Kinase-Glo Luminescent Kinase Assay (Promega, catalog: V6711). SLFN14 (2 μM) and the positive control SUV3 helicase (2 μM) were incubated with ATP (5 μM) along with single-stranded (ss) RNA or ssDNA substrates (2 μM) in ATPase buffer (50 mM Tris pH 7.4, 100 mM NaCl, 1 mM $MgCl_2$, 1 mM TCEP, 0.1 mg/mL BSA, 2% glycerol) for 1 hour at room temperature. See Supplementary Data 2 and Supplementary Data 3 for substrate sequences. An equal volume of Kinase-Glo reagent was added to each reaction and incubated for 10 minutes at room temperature. Luminescence was measured on a PHERAstar FSX plate reader (BMG Labtech) using PheraStar software version 5.70 R4. Reported ATPase activity is the average and standard deviation of three technical replicates plotted in Excel V16.86. Unprocessed data of the ATP hydrolysis assays are shown in the Source Data file.

Mass Photometry

Macromolecular stoichiometry of purified recombinant SLFN14 protein was determined using a TwoMP mass photometer (Refeyn). Glass coverslips were thoroughly cleaned with Milli-Q water followed by isopropyl alcohol, repeating the process twice, before ending with a Milli-Q water rinse. Samples were diluted using filtered mass photometry buffer (20 mM Tris pH 7.4, 150 or 500 mM NaCl, 1 mM $MgCl_2$, 1 mM TCEP) to a concentration of 50 nM protein. Diluted samples were spun at 21,300 x g for 10 minutes immediately prior to performing mass photometry measurements. The focus was set by drop dilution using 10 µL of mass photometry buffer prior to adding 10 µL of protein sample. AcquireMP software (Refeyn) was used to recorded 60-second movies with a total of 1,500-3,000 counts for an optimal resolution of distinct binding events. The molecular mass was determined with DiscoverMP software (Refeyn) and the MassFerence P1 calibrant (Refeyn, catalog: MP-CON-41033). Measurements were performed in triplicate and the average and standard deviation are reported. Representative plots are illustrated in Supplementary Figure 2 using Prism 10 software. Unprocessed data of the mass photometry analysis are shown in the Source Data file.

Western Blot Analysis

Purified Flag-tagged SLFN14 variants (1.3 µg) were resolved by SDS-PAGE and transferred to Immuno-Blot PVDF LF membrane (Bio-Rad, catalog: 1704274) using a semi-dry Trans-Blot Turbo Transfer System (Bio-Rad). Membranes were blocked in 5% nonfat milk, 1x Tris-buffered saline with 0.1% Tween-20 (TBS-T) for 1 hour at room temperature. Antibodies were prepared in 5% nonfat milk, 1% (w/v) BSA, 1x TBS-T. Membranes were incubated with anti-Flag rabbit polyclonal primary antibody (Sigma, catalog: F7425; Lot: 0000131574, 1:1000) or anti-Hsp70 mouse monoclonal primary antibody (Thermo Scientific, catalog: MA3-007; Lot: YA363807, 1:1000) overnight at 4 °C[16, 17]. Membranes were washed in 1x TBS-T and incubated in anti-mouse HRP conjugated antibody (Sigma, catalog: AP127P; Lot: 3778004, 1:1000) or anti-rabbit HRP conjugated antibody (Jackson ImmunoResearch, catalog: 111-035-003; Lot: 159334, 1:1000) for

1 hour at room temperature[18, 19]. Membranes were washed in 1x TBS-T before adding enhanced chemiluminescence detection reagent (Advansta, catalog: K-12045-D20) and imaged using the ChemiDoc MP imaging system (Bio-Rad) with the chemiluminescence setting. Representative blots are shown of three replicates and uncropped blots are shown in the Source Data file.

Negative Stain Electron Microscopy and Image Processing

Purified SLFN14$^{\Delta CTD}$ variant was diluted to ~10 μg/mL using storage buffer and prepared for negative stain using 2% uranyl acetate (Electron Microscopy Sciences, catalog: 22400-2) and carbon support film EM grids (Electron Microscopy Sciences, catalog: 215-412-8400)[20]. Images were collected on a JEM2100 (JEOL) operated at 200 keV using Direct Electron Imaging Manager Data Collection version 2.1.2118.0 sr2 and 2.2.2123.0. A total of ~70 micrographs were collected under low-dose conditions at a nominal magnification of 40,000×. Underfocussed images (-1-5 μm) were recorded on a DE-12 detector with a pixel size at the specimen level of 1.26 Å. Images were processed with cryoSPARC v4.2.1[8]. A total of 31,639 putative particles were selected from the raw images. Reference-free classification of down-sampled and low-pass-filtered (16 Å) images was used to generate a clean dataset that was further classified. Representative 2D classes are shown in Supplementary Information.

References

1. Pei J, Kim BH, Grishin NV. PROMALS3D: a tool for multiple protein sequence and structure alignments. *Nucleic acids research* **36**, 2295-2300 (2008).

2. Waterhouse AM, Procter JB, Martin DM, Clamp M, Barton GJ. Jalview Version 2--a multiple sequence alignment editor and analysis workbench. *Bioinformatics* **25**, 1189-1191 (2009).

3. Fletcher SJ*, et al.* SLFN14 mutations underlie thrombocytopenia with excessive bleeding and platelet secretion defects. *The Journal of clinical investigation* **125**, 3600-3605 (2015).

4. Polokhov D*, et al.* Novel SLFN14 mutation associated with macrothrombocytopenia in a patient with severe haemorrhagic syndrome. *Orphanet J Rare Dis* **18**, 74 (2023).

5. Stapley RJ, Pisareva VP, Pisarev AV, Morgan NV. SLFN14 gene mutations associated with bleeding. *Platelets* **31**, 407-410 (2020).

6. Malone D, Lardelli RM, Li M, David M. Dephosphorylation activates the interferon-stimulated Schlafen family member 11 in the DNA damage response. *The Journal of biological chemistry* **294**, 14674-14685 (2019).

7. Yan B*, et al.* Multiple PDE3A modulators act as molecular glues promoting PDE3A-SLFN12 interaction and induce SLFN12 dephosphorylation and cell death. *Cell Chem Biol* **29**, 958-969.e955 (2022).

8. Punjani A, Rubinstein JL, Fleet DJ, Brubaker MA. cryoSPARC: algorithms for rapid unsupervised cryo-EM structure determination. *Nature methods* **14**, 290-296 (2017).

9. Scheres SH. RELION: implementation of a Bayesian approach to cryo-EM structure determination. *Journal of structural biology* **180**, 519-530 (2012).

10. Kucukelbir A, Sigworth FJ, Tagare HD. Quantifying the local resolution of cryo-EM density maps. *Nature methods* **11**, 63-65 (2014).

11. Jo U, Pommier Y. Structural, molecular, and functional insights into Schlafen proteins. *Exp Mol Med* **54**, 730-738 (2022).

12. Høie MH*, et al.* NetSurfP-3.0: accurate and fast prediction of protein structural features by protein language models and deep learning. *Nucleic acids research* **50**, W510-w515 (2022).

13. Veno ST, Witt MB, Kulikowicz T, Bohr VA, Stevnsner T. Regulation of the human Suv3 helicase on DNA by inorganic cofactors. *Biochimie* **108**, 160-168 (2015).

14. Metzner FJ, Wenzl SJ, Kugler M, Krebs S, Hopfner KP, Lammens K. Mechanistic understanding of human SLFN11. *Nature communications* **13**, 5464 (2022).

15. Kugler M, Metzner FJ, Witte G, Hopfner KP, Lammens K. Phosphorylation-mediated conformational change regulates human SLFN11. *Nature communications* **15**, 10500 (2024).

16. Bowman CJ, Ayer DE, Dynlacht BD. Foxk proteins repress the initiation of starvation-induced atrophy and autophagy programs. *Nature cell biology* **16**, 1202-1214 (2014).

17. Horikawa I*, et al.* Autophagic degradation of the inhibitory p53 isoform Delta133p53alpha as a regulatory mechanism for p53-mediated senescence. *Nature communications* **5**, 4706 (2014).

18. Angage D*, et al.* A broadly cross-reactive i-body to AMA1 potently inhibits blood and liver stages of Plasmodium parasites. *Nature communications* **15**, 7206 (2024).

19. Van den Bossche V*, et al.* PPARalpha-mediated lipid metabolism reprogramming supports anti-EGFR therapy resistance in head and neck squamous cell carcinoma. *Nature communications* **16**, 1237 (2025).

20. Booth DS, Avila-Sakar A, Cheng Y. Visualizing proteins and macromolecular complexes by negative stain EM: from grid preparation to image acquisition. *Journal of visualized experiments : JoVE*, (2011).
