# Supplementary material for: CryoEM structure of the SLFN14 endoribonuclease reveals insight into RNA binding and cleavage
Source: Nat Commun. 2025 Jul 1;16:5848. doi: 10.1038/s41467-025-61091-8 (PMC12215978; doi:10.1038/s41467-025-61091-8)
Supplement: Supplementary file 2 — Description of Additional Supplementary Files [file 41467_2025_61091_MOESM2_ESM.pdf]

Description of Additional Supplementary Files

Supplementary Data 1: Description of protein expression plasmids used in this study.

Supplementary Data 2: RNA sequences used in this study.

Supplementary Data 3: DNA sequences used in this study.

Supplementary Movie 1: CryoEM reconstruction with state 1 SLFN14•RNA model.

Supplementary Movie 2: SLFN14•RNA state 1 and state 2 cryoEM reconstructions.

Supplementary Movie 3: Molecular architecture of the SLFN14 RNA cleft.
