# Supplementary material for: CryoEM structure of the SLFN14 endoribonuclease reveals insight into RNA binding and cleavage
Source: Nat Commun. 2025 Jul 1;16:5848. doi: 10.1038/s41467-025-61091-8 (PMC12215978; doi:10.1038/s41467-025-61091-8)
Supplement: Supplementary file 9 — Reporting Summary [file 41467_2025_61091_MOESM9_ESM.pdf]

nature portfolio

Corresponding author(s): Monica C. Pillon

Last updated by author(s): May 26, 2025

Reporting Summary

Nature Portfolio wishes to improve the reproducibility of the work that we publish. This form provides structure for consistency and transparency in reporting. For further information on Nature Portfolio policies, see our Editorial Policies and the Editorial Policy Checklist.

Statistics

For all statistical analyses, confirm that the following items are present in the figure legend, table legend, main text, or Methods section.

n/a	Confirmed	
☐	☒	The exact sample size (*n*) for each experimental group/condition, given as a discrete number and unit of measurement
☐	☒	A statement on whether measurements were taken from distinct samples or whether the same sample was measured repeatedly
☒	☐	The statistical test(s) used AND whether they are one- or two-sided<br>*Only common tests should be described solely by name; describe more complex techniques in the Methods section.*
☒	☐	A description of all covariates tested
☒	☐	A description of any assumptions or corrections, such as tests of normality and adjustment for multiple comparisons
☐	☒	A full description of the statistical parameters including central tendency (e.g. means) or other basic estimates (e.g. regression coefficient) AND variation (e.g. standard deviation) or associated estimates of uncertainty (e.g. confidence intervals)
☒	☐	For null hypothesis testing, the test statistic (e.g. *F*, *t*, *r*) with confidence intervals, effect sizes, degrees of freedom and *P* value noted<br>*Give P values as exact values whenever suitable.*
☒	☐	For Bayesian analysis, information on the choice of priors and Markov chain Monte Carlo settings
☒	☐	For hierarchical and complex designs, identification of the appropriate level for tests and full reporting of outcomes
☒	☐	Estimates of effect sizes (e.g. Cohen's *d*, Pearson's *r*), indicating how they were calculated

Our web collection on statistics for biologists contains articles on many of the points above.

Software and code

Policy information about availability of computer code

Data collection

CryoEM data was collected with Serial EM version 3.8.16 and EPU 3 software. Negative stain EM was collected with Direct Electron Imaging Manager Data Collection version 2.1.2118.0 sr2 and Direct Electron Imaging Manager Data Collection version 2.2.2123.0. Luminescence was recorded using PheraStar Software version 5.70 R4 and differential scanning fluorimetry was collected using QuantStudio Real-Time PCR Software v1.3. Mass photometry data was recorded using AcquireMP software (Refeyn).

Data analysis

MotionCor2, CryoSPARC v4.2.1 and v.4.6.2, Relion 4.0, CTFFIND4, and DeepEMhancer 0.13 were used for cryoEM image processing and structure determination.
Phenix 1.21-1-5286 and Coot 0.9.8.94 were used for model building, refinement and validation.
UCSF Chimera 1.17.3, UCSF Chimera X 1.8, and Pymol V3.1.3 were used for visual presentation of the CryoEM maps and models.
Excel V16.86 and Prism 10 was used for graph generation.
Jalview 2.11.4.1 and PROMALS3D were used for protein sequence alignment and visualization.
ChemDraw 23.1.2 was used for 2D chemical drawing.
NetSurfP-3.0 was used to calculate relative surface accessibility.
AlphaFold3 was used to generate a tRNASer-bound SLFN14 prediction model.
Protein Thermal Shift Software v1.3 was used to calculate protein melting temperatures.
Proteome Discoverer 2.0 software and Mascot algorithm v2.5 were used for protein identification by LC-MS/MS.
Mass photometry data was analyzed using DiscoverMP software (Refeyn) and Prism 10 software.

For manuscripts utilizing custom algorithms or software that are central to the research but not yet described in published literature, software must be made available to editors and reviewers. We strongly encourage code deposition in a community repository (e.g. GitHub). See the Nature Portfolio guidelines for submitting code & software for further information.

Data

Policy information about availability of data

All manuscripts must include a data availability statement. This statement should provide the following information, where applicable:

- Accession codes, unique identifiers, or web links for publicly available datasets
- A description of any restrictions on data availability
- For clinical datasets or third party data, please ensure that the statement adheres to our policy

All data is included in the manuscript figures, supplemental information, and public repositories. Source data are provided with this paper for raw data files. The cryoEM maps and the atomic coordinates generated in this study have been deposited in the EMDB and PDB under accession codes EMD-49946 (https://www.ebi.ac.uk/pdbe/entry/emdb/EMD-49946), EMD-49947 (https://www.ebi.ac.uk/pdbe/entry/emdb/EMD-49947), and PDB 9NYY (http://doi.org/10.2210/pdb9NYY/pdb). Previously published accession codes are deposited in the PDB as follows PDB 5YD0 (http://doi.org/10.2210/pdb5YD0/pdb), 7LRD (http://doi.org/10.2210/pdb7LRD/pdb), 9GMW (http://doi.org/10.2210/pdb9GMW/pdb), 9GMX (http://doi.org/10.2210/pdb9GMX/pdb), 7Q3Z (http://doi.org/10.2210/pdb7Q3Z/pdb), 2B0D (http://doi.org/10.2210/pdb2B0D/pdb), 2BAM (http://doi.org/10.2210/pdb2BAM/pdb), 1CL8 (http://doi.org/10.2210/pdb1CL8/pdb), 1QPS (http://doi.org/10.2210/pdb1QPS/pdb), 7ZEL (http://doi.org/10.2210/pdb7ZEL/pdb), and 9ERF (http://doi.org/10.2210/pdb9ERF/pdb). The mass spectrometry proteomics data have been deposited to the ProteomeXchange Consortium via the PRIDE partner repository with the dataset identifier PXD064237 (http://proteomecentral.proteomexchange.org/cgi/GetDataset?ID=PXD064237). Source data are provided with this paper.

Research involving human participants, their data, or biological material

Policy information about studies with human participants or human data. See also policy information about sex, gender (identity/presentation), and sexual orientation and race, ethnicity and racism.

Reporting on sex and gender	Reporting on sex and gender is not relevant as there are no human participants, their data, or biological material in this study.
Reporting on race, ethnicity, or other socially relevant groupings	Reporting on race, ethinicity, or other social relevant groupings is not relevant as there are no human participants, their data, or biological material in this study.
Population characteristics	Population characteristics is not relevant to this study as there are no human participants, their data, or biological material in this study.
Recruitment	Recruitment is not relevant to this study as there are no human participants, their data, or biological material in this study.
Ethics oversight	Ethics oversight is not relevant to this study as there are no human participants, their data, or biological material in this study.

Note that full information on the approval of the study protocol must also be provided in the manuscript.

Field-specific reporting

Please select the one below that is the best fit for your research. If you are not sure, read the appropriate sections before making your selection.

☒ Life sciences ☐ Behavioural & social sciences ☐ Ecological, evolutionary & environmental sciences

For a reference copy of the document with all sections, see nature.com/documents/nr-reporting-summary-flat.pdf

Life sciences study design

All studies must disclose on these points even when the disclosure is negative.

Sample size	CryoEM: From three grids prepared with three independent biological replicates, three data collection sessions (two sessions without stage tilt and one with stage tilt) resulted in a total of 11,067 movies. Particles (10,302,578) were picked from all the movies. 2D and 3D classification seperated intact particles for refinement steps where a total of 338,469 and 188,010 particles contributed to the final map for state 1 and state 2, respectively. This sampling was sufficient to cover a complete distribution of viewing angles.<br>In vitro assays (e.g. RNA binding, RNA cleavage, DSF, ATP hydrolysis, mass photometry): As is standard practice, at least three biological replicates were performed for each condition.<br>Size Exclusion Chromatography analysis: At least two biological replicates of SLFN14 variants were prepared and analyzed.
Data exclusions	During cryoEM data processing poor micrographs and bad particles were discarded following 2D and 3D classification.
Replication	Each cryoEM map was created from three combined datasets. We determined two cryoEM reconstructions of SLFN14, which all yielded similar 3D maps with variability in the periphery of the particle. All nuclease assays, EMSAs, Northern blots, Western Blot, mass photometry analysis, differential scanning fluorimetry assays, and ATPase assays were performed with at least three technical replicates from multiple independent protein purifications. All attempts at replication were successful.
Randomization	SLFN14 particles were randomly selected using Relion 4.0 and CryoSparc v4.2.1 for reference-free 2D classification.

Blinding	Blinding was not relevant to this study as there are no experiments subject to bias.

Reporting for specific materials, systems and methods

We require information from authors about some types of materials, experimental systems and methods used in many studies. Here, indicate whether each material, system or method listed is relevant to your study. If you are not sure if a list item applies to your research, read the appropriate section before selecting a response.

Materials & experimental systems

n/a	Involved in the study
☐	☒ Antibodies
☐	☒ Eukaryotic cell lines
☒	☐ Palaeontology and archaeology
☒	☐ Animals and other organisms
☒	☐ Clinical data
☒	☐ Dual use research of concern
☒	☐ Plants

Methods

n/a	Involved in the study
☒	☐ ChIP-seq
☒	☐ Flow cytometry
☒	☐ MRI-based neuroimaging

Antibodies

Antibodies used	Primary antibodies described in the Supplementary Methods section:<br>anti-FLAG (used 1:1000, Sigma, catalog: F7425, Lot: 0000131574) - mentioned in supplementary ref. 16<br>HSP70 antibody (used 1:1000, Thermo, catalog: MA3-007, Lot: YA363807) - mentioned in supplementary ref. 17<br>Secondary antibodies described in the Supplementary Methods section:<br>anti-mouse IgG HRP conjugated (used 1:1000, EMD Millipore, catalog: AP127P, Lot: 3778004) - mentioned in supplementary ref. 18<br>anti-rabbit IgG HRP conjugated (used 1:1000, Jackson ImmunoResearch, catalog: 111-035-003, Lot: 159334). - mentioned in supplementary ref. 19
Validation	This study only used commercial antibodies that are widely used in the field and were validated by the manufacturer for Western blot applications. All commercial antibodies were validated using cells not expressing the protein under study.

Eukaryotic cell lines

Policy information about cell lines and Sex and Gender in Research

Cell line source(s)	Hek293F (Thermo Scientific, catalog: R79007)
Authentication	Commercial cell line was not authenticated.
Mycoplasma contamination	Cell line was not tested for mycoplasma contamination.
Commonly misidentified lines<br>(See ICLAC register)	No commonly misidentified cell lines were used in this study.

Plants

Seed stocks	Seed stocks is not relevant to this study because plants were not used.
Novel plant genotypes	Novel plant genotypes is not relevant to this study because plants were not used.
Authentication	Authentication is not relevant to this study as plants were not used.
